# Foxp3 enhances HIF-1α target gene expression in human bladder cancer through decreasing its ubiquitin-proteasomal degradation

**DOI:** 10.18632/oncotarget.11395

**Published:** 2016-08-19

**Authors:** Yeong-Chin Jou, Yuh-Shyan Tsai, Chang-Te Lin, Chun-Liang Tung, Cheng-Huang Shen, Hsin-Tzu Tsai, Wen-Horng Yang, Hung-I Chang, Syue-Yi Chen, Tzong-Shin Tzai

**Affiliations:** ^1^ Department of Urology, Chia-Yi Christian Hospital, Chia-Yi, Taiwan; ^2^ Department of Urology, National Cheng Kung University Hospital, College of Medicine, National Cheng Kung University, Tainan, Taiwan; ^3^ Department of Pathology, Chia-Yi Christian Hospital, Chia-Yi, Taiwan; ^4^ Department of Medical Researh, Chia-Yi Christian Hospital, Chia-Yi, Taiwan; ^5^ Department of Urology, School of Medicine, China Medical University, Taichung, Taiwan; ^6^ Department of Urology, Tainan Municipal An-Nan Hospital, China Medical University, Tainan, Taiwan

**Keywords:** bladder neoplasms, Foxp3, immunohistochemistry, prognosis, glycolysis

## Abstract

Hypoxia-inducible factor-1α (HIF-1α) can control a transcriptional factor forkhead box P3 (Foxp3) protein expression in T lymphocyte differentiation through proteasome-mediated degradation. In this study, we unveil a reverse regulatory mechanism contributing to bladder cancer progression; Foxp3 expression attenuates HIF-1α degradation. We first demonstrated that Foxp3 expression positively correlates with the metastatic potential in T24 cells and can increase the expression of HIF-1α-target genes, such as vascular endothelial growth factor (VEGF) and glucose transporter (GLUT). Foxp3 protein can bind with HIF-1α, particularly under hypoxia. *In vivo* ubiquination assay demonstrated that Foxp3 can decrease HIF-1α degradation in a dose-dependent manner. Knocking-down of Foxp3 expression blocks *in vivo* tumor growth in mice and prolongs mice's survival, which is associated with von Willebrand factor expression. Thirty-three of 145 (22.8 %) bladder tumors exhibit Foxp3 expression. Foxp3 expression is an independent predictor for disease progression in superficial bladder cancer patients (*p* = 0.032), associated with less number of intratumoral CD8^+^ lymphocyte. The metaanalysis from 2 published datasets showed Foxp3 expression is positively associated with GLUT−4, −9, and VEGF-A, B-, D expression. This reverse post-translational regulation of HIF-1α protein by Foxp3 provides a new potential target for developing new therapeutic strategy for bladder cancer.

## INTRODUCTION

Bladder cancer is the fifth most common cancer among men all over the world [1]. Approximately 70-80% of bladder cancers were superficial diseases at initial diagnosis and most of them (about 70 %) easily recur and about 15% may progress as muscle-invasive disease despite adjuvant intravesical chemotherapy or immunotherapy [2]. Although a standard biomarker for the prediction of clinical outcome is still lacking, many conventional tumor markers and molecular pathways involved in these cancers or treatment outcomes have been investigated [3–5]. For example, fibroblast growth factor receptor (FGFR), vascular endothelial growth factor (VEGF), epidermal growth factor receptor (EGFR), or p53 have been reported to play a pivotal role in tumor recurrence and progression of bladder cancer [2, 6]. Hypoxia-inducible factor-1 alpha (HIF-1α) protein expression is an unfavorable prognostic factor in bladder urothelial carcinoma as it can up-regulate FGFR3, VEGF, and GLUT-1 which contribute to tumor cell survival, tumor angiogenesis, and aerobic glycolysis in response to tumor hypoxia [7–9]. Recently, several large-scale promising genomic studies of bladder cancer showed bladder cancers can split into three pan-cancer subtypes, which is unlike other human malignancies [10], although TP53, ErbB2, phosphatidylinositol-3-OH kinase/AKT/mTOR pathways are the major genomic alternation in bladder cancer as other human malignancies [11]. Also, results of a phase 1 study of the anti-programmed death ligand-1(PD-L1) monoclonal antibody (immune checkpoint inhibitor) have shown rapid and ongoing responses in metastatic bladder cancer, particularly in patients with intratumoral PD-L1-expressing T lymphocytes [12]. Therefore, it is important to understand the tumor immunology in bladder cancer patients for more precision therapy.

The forkhead box P3 (Foxp3) is an X-linked transcription factor that is required for induction of the immunosuppressive functions in regulatory T lymphocytes [13]. Although its expression was first considered to be specific to this cell type, studies have demonstrated that Foxp3 protein is expressed as a marker for regulatory T cells (Treg) cells and as an onco-suppressor in several mice models, including breast, prostate, and pancreatic cancers via either as a transcriptional repressor of c-Myc, Skp2, and HER2 gene expression, or a regulator of interleukin (IL)−6 or −8 expression, respectively [14–16]. Further, Foxp3 expression in Treg cells can be regulated through HIF-1α-mediated proteasomal degradation under hypoxic and normoxic condition, which provides the cues between metabolic stress and immune system [17]. Foxp3 expression in Treg and effector T cells influences glucose transporter-1 (GLUT-1) expression through inhibiting Akt phosphorylation, providing the molecular basis of the link between Foxp3-related immune system and glucose metabolism [18]. In contrast, the majority of clinical studies regarding the role of Foxp3 expression in human malignancies demonstrated its unfavorable role and correlation with lymph node or visceral metastases, including non-small cell lung cancer [19, 20], and gastric cancer [21]. In invasive human bladder cancer, Foxp3 expression is a worse prognostic factor for overall survival [22, 23], in which the presence of Foxp3Δ3 isoform protein may contribute to *in vitro* spheroid formation in SW780 cells and larger tumor growth in mice, as well as chemoresistance [23]. Despite this, the underlying mechanism remains unclear.

In the present study we demonstrated that the Foxp3 can enhance HIF-1α protein expression through decreasing ubiquitin-mediated proteasomal degradation in bladder cancer cells and influences VEGF signaling and intratumoral immunity, contributing an unfavorable prognosis in bladder cancer.

## RESULTS

### Foxp3 expression upregulates *GLUT*, and *VEGF* mRNA expression and is associated with aerobic glycolysis

**Figure 1:**
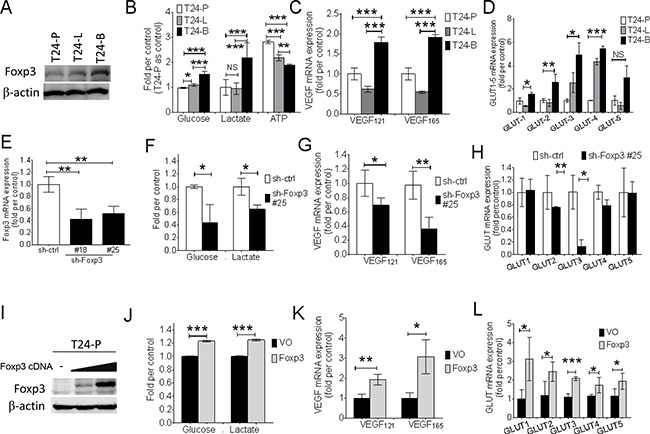
Foxp3 expression is associated with aerobic glycolysis and VEGF, glucose transporter expression **A.** Foxp3 expression is examined with western blotting assay in three T24 bladder cancer sublines (T24-P, T24-L, and T24-B). **B.** Assays for glucose, lactate, and ATP production in three T24 sublines. **C.** VEGF mRNA and **D.** GLUT-1~5 mRNA expression is examined in three T24 sublines using qRT-PCR assay. **E.** Foxp3 mRNA expression is examined in Foxp3-knocking down T24-B transfectants and its control. **F.** Assays for glucose and lactate production, **G.** VEGF mRNA expression, and **H.** GLUT-1~5 as assayed in Foxp3-knocking down transfectant #25 and its control. **I.** Foxp3 expression is examined with western blotting assay after transient transfection of Foxp3 plasmid into T24-P bladder cancer cells. **J.** Assays for glucose and lactate production, **K.** VEGF mRNA expression, and **L.** GLUT-1~5 as assayed in pooled Foxp3-overexpressing T24-P transfectants and its control. T24-P, T24 parental subline; T24-L, metastatic lung T24 subline; T24-B, metastatic bone T24 subline; ATP, Adenosine triphosphate; GLUT, glucose transporter; EGFR, epidermal growth factor receptor; VEGF, vascular endothelial growth factor; NS, not significant; *, p<0.05; **, p<0.01, ***, p<0.001. Data are represented as mean ± SD.

Three T24 sublines were used to explore whether the association between Foxp3 expression and aerrobic glycolysis exists or not. Western blotting result showed the metastatic T24-B subline expressed more Foxp3 protein than the other two sublines-the parental vector control (T24-P) and metastatic lung one (T24-L) (Figures 1A). The T24-B subline exhibited more glucose, lactate, and less ATP amounts than the other two sublines, which met with Warburg effect (Figure 1B), as well as higher *VEGF_121_*, VEGF*_165_* and four *GLUT* member mRNA expressions (Figure 1C and 1D). After knocking-down Foxp3 expression in T24-B cells, glucose content and lactate production declined (Figure 1F). Both *VEGF_121, 165_*, and *GLUT-3,-4* mRNA expression were down-regulated (Figure 1G and 1H), as well as GLUT-3 and EGFR protein in the western blotting assay (Supplementary Figure S1). In contrast, both increased amount of glucose content and lactate production increased, and up-regulation of *VEGF* and *GLUT* mRNA were detected in the T24-P cells with ectopic expression of Foxp3. (Figures 1I-1L).

### Foxp3 enhances HIF-1α expression through decreasing ubiquitin-proteasomal degradation

**Figure 2:**
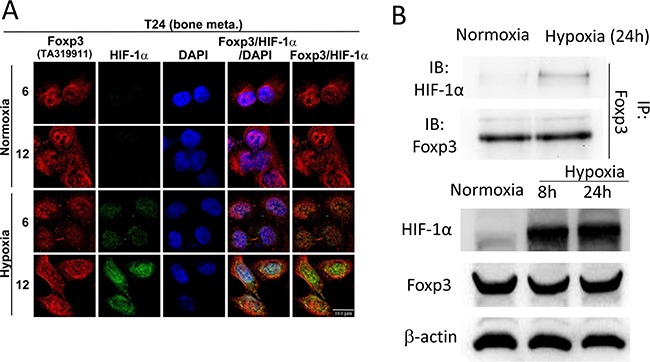
Foxp3 can bind with HIF-1α in hypoxia condition **A.** T24-B cell were stained with anti-Foxp3, anti-HIF-1α and DAPI for confocal microscopy studies in normoxic and hypoxic circumstance as indicated times. Scale bars, 10 μm. **B.** T24-B cell were maintained in normoxic and hypoxic circumstance as indicated times. Cell lysates were immunoprecipitated with anti-Foxp3 antibody and subjected to Foxp3 and HIF-1α western blot.

**Figure 3:**
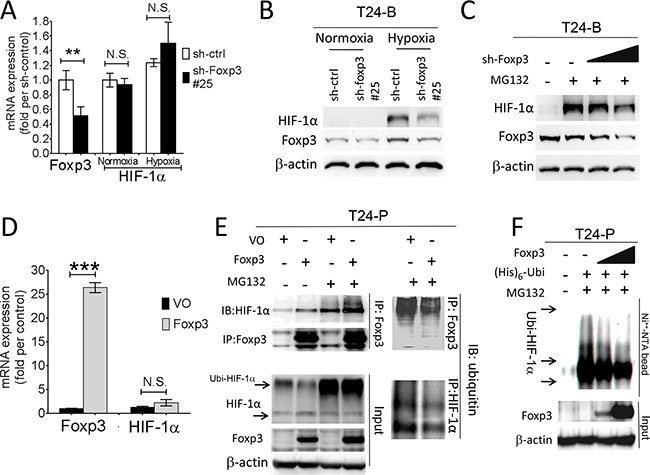
Foxp3 expression decreased ubiquitin-proteasomal degradation of HIF-1α protein **A.** HIF-1α mRNA expression were examined in T24-B Foxp3 knocking down cells and its control both in normoxic and hypoxic circumstances using qRT-PCR assays. **B.** Foxp3-knocking down T24B cells and its control were maintained in normoxic and hypoxic circumstance and cell lysates for harvested for western blot. **C.** After transfection, cells were maintained in normoxic and hypoxic circumstance and treated with MG132 (10μM) for 24 hours before harvesting for western blotting assay. **D.** Foxp3 and HIF-1α mRNA expression were examined in Foxp3-overexpressing T24-P cells and its control in normoxic circumstance using qRT-PCR assays. **E.** T24-P cells were transfected with Foxp3 cDNA-containing plasmids or control as indicated conditions with or without MG132 treatment. Cell lysates were immunoprecipitated with anti-Foxp3 or anti-ubiquitin antibody and subjected to Foxp3, HIF-1α or ubiquitin western blot. **F.** *In vivo* ubiquitination assay. T24-P cells were transfected with Foxp3 cDNA-carrying plasmid in a dose-dependent manner, as well as histdine-tagged ubiquitin cDNA. Cell lysates were harvested and Histiidine-containing protein complex were pulled down using Ni^++^-NTA magnetic bead for subquentent immunoblotting assay. The expression of β-actin used as a loading control. NS, not significant; *, p<0.05; **, p<0.01, ***, p<0.001. Data are represented as mean ± SD.

The physical interaction between Foxp3 and HIF-1α plays a pivotal role in the differentiation of regulatory/T(H)17 immune cells [17]. Since Foxp3 expression can increase HIF-1α target gene expression in T24 and its sublines, we further investigated the role of Foxp3 expression in HIF-1α regulation. First, T24-B and HeLa (a cervical cancer cell line) cells were investigated with double immunoflorescence confocal microscopic studies (Figures 2A and Supplementary Figure S2). In both cells, Foxp3 protein can be visualized regardless oxygen deprivation or not; however, HIF-1α can only be visualized in the nucleus under hypoxic condition rather than normoxic condition due to a short half-life. The physical binding between Foxp3 and HIF-1α can be detected in the cell nucleus of T24-B cells and HeLa cels under hypoxia, as shown in the immunoprecipitation study (Figure 2B). While knocking down Foxp3 expression in T24-B cells, the level of HIF-1α mRNA did not change regardless in normoxic or hypoxic conditions (Figure 3A) and HIF-1α protein expression is reduced under hypoxic condition (Figure 3B) or when cells treated with proteasome inhibitor MG132 (Figure 3C). On the other hand, while ectopic overexpression of Foxp3 protein in T24-P cells, the HIF-1α protein increased (Figure 3E) and the level of HIF-1α mRNA did not change (Figure 3D). The *in vivo* ubiquitination assay showed Foxp3 overexpression can decrease ubiquitination of HIF-1α protein in a dose-dependent manner (Figure 3E and 3F). Taken together, Foxp3 can be detected to be able to bind with HIF-1α protein, particularly upon hypoxia or MG132 treatment and enahnce HIF-1α protein expression through decreasing ubiquitin-mediated proteasomal degradation in human bladder cancer cells.

### Knocking-down of Foxp3 expression blocks *in vivo* tumor growth in mice

**Figure 4:**
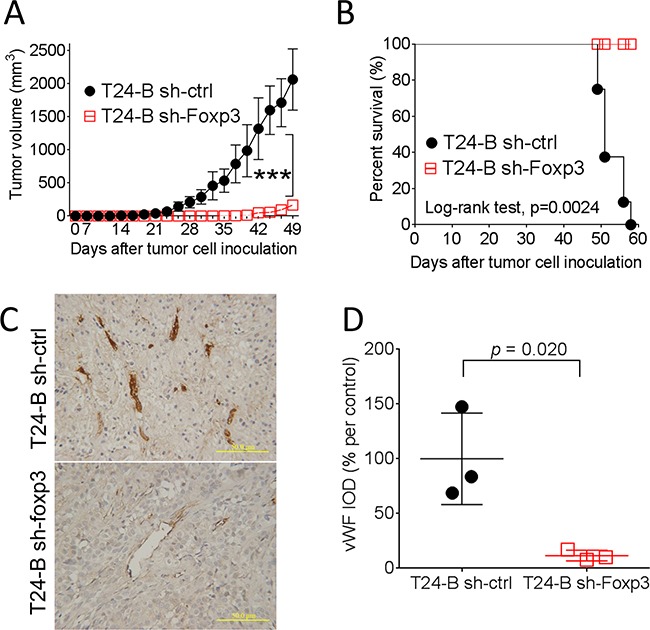
Effect of Foxp3 expression on bladder tumor growth in mice Female NOD-SCID mice were subcutaneously injected with 1×10^6^ Foxp3 knocking-down T24-B in 100 ml serum-free medium or its control. *In vivo* growth **A.** and survival curve **B.** were record and analyzed. **C.** The growing tumor were harvested at day 58 for immunostaining of *von Willebrand factor*, Magnification × 200. Scale bars, 50 μ. **D.** The integrated density were calculated using Imaging-Pro Plus software and compared with unpaired *t*-test.

To investigate *in vivo* biological effect of Foxp3 expression in mice, female NOD-SCID mice were injected in the flank area subcutaneously with Foxp3 knocking-down T24-B cells or the control. The results demonstrated that knocking-down of Foxp3 expression blocks *in vivo* tumor growth of T24-B cells in mice and prolongs the survival (*p* values, < 0.0001 and 0.0024, respectively) (Figure 4A and 4B). Consistently, the density of von Willebrand factor (vWF) immunostaining is decreased in the Foxp3-knokcking down tumor xenografts, as compared with the control (*p* = 0.020) (Figure 4C and 4D).

### Foxp3 protein expression in human bladder cancer

**Figure 5:**
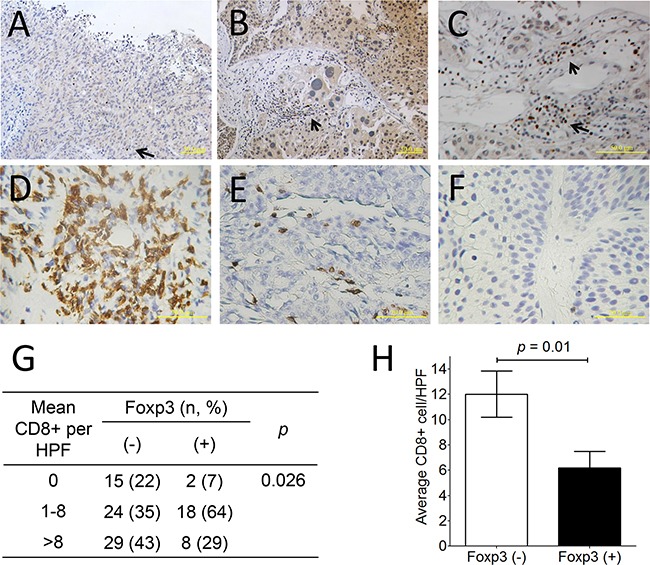
Foxp3 protein expression and CD8+ lymphocyte in human bladder cancer **A.** Negative Foxp3 expression (× 200; scale bar, 50 μm), **B.** Positive Foxp3 expression (× 200; scale bar, 50 μm), **C.** Foxp3+ lymphocytes within the bladder tumor (× 400; scale bar, 50 μm). **D.** More than 8 CD8+ lymphocyte within bladder tumors, **E.** 1≤ CD8+ lymphocyte number ≤8, and **F.** none of CD8+ lymphocytes based on the average number determined from 10 random 0.0328-mm^2^ digital images captured under high power field (× 320; scale bar, 20 μm). **G.** Distribution and mean number of CD8+TILs from 96 bladder cancer specimen according to Foxp3 expression. **H.** The comparison of average CD8+ TILs number between Foxp3-expressing tumors or not is analyzed using unpaired *t*-test. Foxp3-expressing lymphocytes (arrow).

Immunohistochemically, thirty-three of 145 (22.8 %) bladder tumors expressed Foxp3 protein (Figure 5A-5C). Overall, there were 16 of 98 (16.3%) male and 17 of 47 (36.2%) female patients exhibiting Foxp3-expressing tumors in this cohort (*p* = 0.008, chi-squared test) (Supplementary Table S1), as well as in patients with superficial tumors (p < 0.0001) (Table 1). Except for the gender factor, there was lack of any association between Foxp3 expression and other clinicopathological factors, including age, history of urothelial carcinoma, and tumor number, morphology, grade, and pathological stage (all *p* values > 0.05) (Supplementary Table S1). Foxp3-expressing TILs were found only in 10 tumors regardless of Foxp3-expressing tumors or not (Figure 5C), so that it was not further analyzed. Totally, 96 section slides were available for CD8 immunostaining, including 68 Foxp3 (−) and 28 Foxp3 (+) tumors. CD8^+^ lymphocytes can be detected within tumors in variable numbers (Figure 5D-5F). Tumors with Foxp3 expression exhibited less average number of CD8^+^TILs than did those without Foxp3 expression (*p*=0.01, unpaired t-test) (Figure 5H), as well as less frequency of higher CD8^+^ cells density (*p*=0.026, chi-square test) (Figure 5G).

**Table 1:** Clinicopathological correlate of Foxp3 expression in superficial bladder carcinoma tissues

	Total	Fox p3(−)	Fox p3(+)	*p* value
Total, n	115	87	28	
Age, (yr), mean ± SD	67.6 ± 12.8	67.7 ± 13.4	67.4 ± 11.3	0.915
Median	70	71	68	
25~75 percentile	58~78	58~78	60~76	
Gender				
Male	80	70	10	< 0.0001
Female	35	17	18	
History of UC				
Primary	62	45	17	0.407
Recurrence	53	42	11	
ESRD				
No	95	72	23	0.832
Yes	20	15	5	
Multiplicity				
Single	41	31	10	0.994
Multiple	74	56	18	
Tumor grade				
Low	16	11	5	0.534
High	99	76	23	
Stage				
Ta	56	42	14	0.874
T1	59	45	14	
Intravesical chemotherapy				
No	47	32	15	0.116
Yes	68	55	13	
Recurrence				
No	35	24	11	0.435
Yes	70	53	17	
Progression to T2				
No	98	79	19	0.008
Yes	17	8	9	
Death				
No	96	74	22	0.609
Yes	19	13	6	

UC, urothelial carcinoma

### Prognostic values of Foxp3 expression in superficial bladder tumors

Since the majority of tumors in this cohort were superficial, we further analyzed the prognostic values of Foxp3 expression in superficial tumors. Sixty-eight of 115 (59.1%) patients received post-TURBT intravesical therapy, including 49 epirubicin, 16 mitomycin-C, and 3 BCG. During follow-up (median, 48 months), there were 70 (60.9 %) recurrences, 17 (14.7%) progressions, and 19 (16.5%) death (Supplementary Table S1). Patients with Foxp3-expressing tumors had higher frequency of disease progression to muscle invasiveness than did those without Foxp3-expressing tumors (*p* = 0.008, chi-square test) (Table 1). There were no correlation between Foxp3 expression and the other two outcomes (recurrence and non-specific death, *p* = 0.435 and 0.609, respectively).

**Figure 6:**
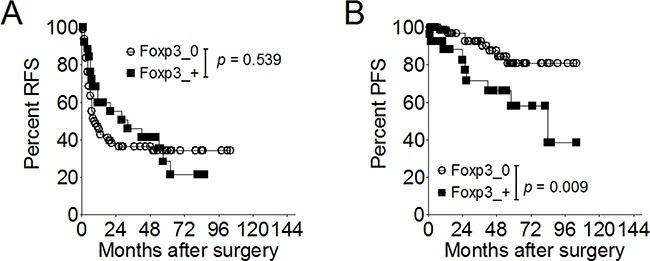
Effect of Foxp3 expression on disease recurrence or progression in 115 patients with superficial bladder cancer **A.** Proportion of recurrence-free survival according to the Foxp3 expression. **B.** Proportion of progression-free survival according to the Foxp3 expression. RFS, recurrence-free survival; PFS, progression-free survival.

In terms of tumor recurrence, urivariate analyses demonstrated that tumor stage is significantly associated with RFS [HR, 1.91; 95% confidence interval (CI), 1.18-3.07; *p* = 0.008], and tumor grade is a borderline significant predictor (HR, 2.12; 95% CI, 0.97-4.64; *p* = 0.060). Neither intravesical therapy (*p* = 0.694) nor Foxp3 expression was a significant predictor for tumor recurrence (*p* = 0.539) (Figure 6A). Multivariate analysis showed tumor stage is an independent prognostic factor for tumor recurrence (HR, 1.76; 95% CI, 1.08-2.85; *p* = 0.023) (Table 2).

**Table 2:** Univariate and multivariate analyses of variables associated with recurrence and progression in 115 superficial bladder cancer patients

Variables	RFS		PFS	
	HR (95% CI)	*p*	HR (95% CI)	*p*
*Univariate analysis*				
Gender	0.67 (0.39-1.15)	0.144	2.05 (0.79-5.37)	0.142
History of UC	1.45 (0.91-2.32)	0.122	1.48 (0.57-3.85)	0.418
ESRD	0.87 (0.46-1.66)	0.672	0.90 (0.26-3.13)	0.862
Multiplicity	1.27 (0.77-2.11)	0.350	3.36 (0.77-14.7)	0.108
Grade	2.12 (0.97-4.64)	0.060	26.8 (0.10-6590)	0.242
Stage	1.91 (1.18-3.07)	0.008	2.79 (1.02-7.67)	0.046
Intravesical therapy	0.91 (0.57-1.46)	0.694	0.23 (0.08-0.67)	0.007
Foxp3 expression	0.84 (0.49-1.46)	0.539	3.33 (1.28-8.63)	0.009
*Multivariate analysis*^2^				
Grade	1.81 (0.81-4.01)	0.146	-	-
Stage	1.76 (1.08-2.85)	0.023	-	-
Intravesical therapy	-	-	0.29 (0.09-0.89)	0.031
Foxp3 expression	-	-	3.14 (1.10-8.91)	0.032

In term of disease progression, univariate analyses demonstrated that tumor stage, intravesical therapy, and Foxp3 expression were significantly associated with PFS (Table 2) (Figure 6B). Multivariate analysis showed intravesical therapy was an independent prognostic predictors for disease progression [HR, 0.29; 95% CI, 0.09-0.89; *p*=0.031), as well as the Foxp3 expression [HR (95% CI), 3.14 (1.10-8.91); *p* = 0.032].

### Foxp3 expression is correlated with GLUT−4, −9, and VEGF-A, -B, -D

**Figure 7:**
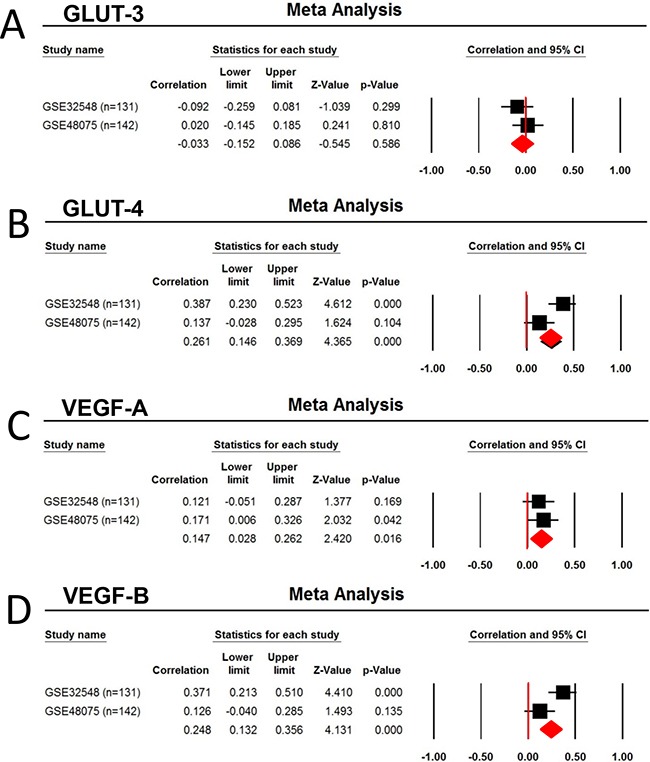
Correlation of Foxp3 expression with the Glucose transporter and VEGF Two human urothelial carcinoma dataset GSE32548 (n=131) and GSE48075 (n=142) were selected for the metaanalysis due to the used same platform (GPL6947, Illumina HumanHT-12 V3.0 expression bead chip) and larger number of patients (more than 100). **A.** GLUT3, **B.** GLUT-4, **C.** VEGF-A, and **D.** VEGF-B.

To obtain the external validation, data mining from 2 published dataset GSE32548 (n=131) and GSE48075 (n=142) demonstrated Foxp3 mRNA expression is significantly associated with GLUT-4 (*r* = 0.261, *p <* 0.0001), GLUT-9 (*r* = 0.269, *p<*0.0001), VEGF-A (*r* = 0.147, *p = 0.0*16), VEGF-B (*r* = 0.248, *p<*0.0001), and GLUT-D (*r* = 0.158, *p =* 0.0009), but not with HIF-1α (*p* > 0.05) (Table 3) (Figure 7).

**Table 3:** Correlation of Foxp3 expression with the Glucose transporter family member: result of a meta-analysis of GSE32548 (n=131) and GSE48075 (n=142)

Gene symbol	ID_REF* (GPL6947)	Correlation	Lower limit	Upper limit	*Z-value*	*p*-value
**Glucose transporter**						
SLC2A1	ILMN_1659027	−0.0005	−0.124	0.114	−0.085	0.933
SLC2A2	ILMN_1755720	−0.086	−0.203	0.034	−1.403	0.161
SLC2A3	ILMN_1775708	−0.033	−0.152	0.086	−0.545	0.586
SLC2A4	ILMN_1782545	0.261	0.146	0.369	4.365	<0.0001
SLC2A5	ILMN_1671337	0.080	−0.093	0.248	0.907	0.365
SLC2A6	ILMN_1778321	−0.008	−0.127	0.111	−0.131	0.896
SLC2A7	ILMN_1707370	−0.029	−0.200	0.143	−0.332	0.740
SLC2A8	ILMN_1724609	0.113	−0.007	0.229	1.852	0.064
SLC2A9	ILMN_1723803	0.269	0.155	0.376	4.506	<0.0001
SLC2A10	ILMN_1663351	−0.046	−0.165	0.073	−0.757	0.449
SLC2A11	ILMN_1748090	0.007	−0.112	0.127	0.122	0.903
SLC2A12	ILMN_1766261	−0.076	−0.193	0.044	−1.239	0.215
**VEGF**						
VEGF-A	ILMN_1803882	0.147	0.028	0.262	2.420	0.016
VEGF-B	ILMN_1772274	0.248	0.132	0.356	4.131	<0.0001
VEGF-C	ILMN_1701204	−0.044	−0.163	0.076	−0.720	0.472
VEGF-D	ILMN_1707612	0.158	0.040	0.273	2.612	0.0009
**HIF-1α**						
HIF-1α	ILMN_2379788	0.011	−0.109	0.130	0.172	0.863
HIF-1α	ILMN_1681283	−0.108	−0.225	0.011	−1.777	0.076

## DISCUSSION

In the present study we demonstrated that Foxp3 expression is an independent predictor for disease progression in superficial bladder cancer patients, which is inversely associated with average number of CD8^+^TILs. *In vitro* assays using three T24 sublines showed Foxp3 expression correlated the metastatic ability and can upregulate certain glucose transporter members, VEGF_121_ and VEGF_165_ mRNA expression through decreasing ubiquitin-mediated proteasomal degradation of HIF-1α protein. The metaanalysis from 2 larger published datasets of bladder cancer showed Foxp3 expression is significantly associated with GLUT−4, −9 and VEGF-A, -B, -D mRNA expression, rather than HIF-1α mRNA expression. These findings support the pivotal role of Foxp3 expression in the disease progression of human bladder cancer through upregulating HIF-1α target gene expression.

Conventionally, Foxp3 protein serves as a transcriptional factor that binds to about 700 genes and regulates their expression [13]. Subcellular localization of Foxp3 protein can be immunoblotted in the nucleus, cytoplasm, or both sites in various human cells. Foxp3 expression can be visualized in the nuclei of regulatory T lymphocytes under HIF-1α regulation [17]. Foxp3 protein can be found in the majority of epithelial nuclei of the normal prostate and in about 30% of prostate cancer tissue samples, where served as a tumor suppressor via transcriptionally repressing *cMYC* gene [15]. Also, results are not consistent in breast cancer tissues [14, 24, 25] and in pancreatic cancer tissues [16], although it can transcriptionally repress both expressions of the HER2 and Skp2 gene in the breast cancer [14]. In term of bladder cancer, Foxp3 expression can be detected in tumor cell nuclei or cytoplasm and a positive Foxp3 expression (either 10 cytoplasmic, 3 nuclear, or 4 mixed) is an unfavorable indicator for disease progression and overall survival [22]. However, the underlying biological function of Foxp3 protein remains unclear in human bladder cancer. Our study further demonstrated that Foxp3 protein can enhance HIF-1α target gene expression (i.e., VEGF, and GLUT) through attenuating HIF-1α protein degradation post-translationally.

CD8^+^TILs has been thought to be a favorable indicator for intratumoral immunity in several human malignancies, including bladder cancer. In a cohort of 69 patients with urothelial carcinoma, Sharma et al emphasized in patients with muscle-invasive or advanced urothelial tumors that higher numbers of CD8^+^TILs within the tumor (≥8 per high power field) is a favorable prognostic factor for disease-free survival and overall survival [26]. Our previous study had demonstrated that preoperative circulating CD8^+^ lymphocytes inversely correlated with CD8^+^TILs and that urothelial tumor patients with higher number of preoperative CD8^+^ circulating lymphocytes had shorter recurrence-free survival than did those with lower ones [27]. Several tumor-derived immunosuppressive factors have been postulated to play an important role in malignant progression, including VEGF, IL-10, transforming growth factor-beta (TGF-β), prostaglandin E_2_, and so on [28]. These tumor-derived factors can dysfunctionalize antigen-presenting cells and enhance regulatory T cells, which suppresses intraturmoral CD4+ and CD8+ T lymphocytes [29]. In the current study, we demonstrated higher number of CD8^+^TILs can be seen in Foxp3-negative tumors, consistent with favorable prognosis. *In vitro* T24 subline assays showed higher Foxp3-expressing T24-B subline exerts higher VEGF_121_ or VEGF_165_ mRNA expression that the other two lower Foxp3-expressing sublines, which can be reversed by knocking-down of Foxp3 expression. Taken together, Foxp3 expression in tumor cells can suppress intratumoral immunity in bladder cancer, which might be mediated though HIF-1α and its downstream VEGF production.

There are only few reports regarding the link between glucose metabolism and intratumoral immunity. Singer, et al reported that the correaltion of GLUT-1 expression and lower number of CD8^+^TILs in clear cell renal cell carcinoma samples [30]. Our data demonstrated both increased aerobic glycolysis (less ATP production despite of increased glucose influx and lactate production) and VEGF production were associated with disease progression in the *in vitro* T24 cell subline model, which can be regulated though Foxp3-mediated HIF-1α expression. Such findings can be exploited for targeted therapy in bladder cancer [31]. Casares et al reported that a small peptide P60 (RDFQSFRKMWPFFAM) can serve as a Foxp3 inhibitor that inhibits regulatory T cell activity and enhances vaccine efficacy in mice [32].

As well as HIF-1β mRNA in cells, HIF-1α mRNA are ubiquitously and constitutively expressed regardless of the level of oxygen tension. The regulation of HIF-1α depends on the protein stability. In normoxia, HIF-1α protein is very unstable with a short half-life of less than 5 min. The von Hippel–Lindau (VHL) protein can target human HIF-1α after a key step of prolyl hydroxylation at residues 402 and 564 and form a E3-ubiquitin ligase complex for ubiquitin-mediated proteasomal degradation [33]. Several molecules can either positively or negatively regulate HIF-1α expression or activity, mainly via protein-protein interaction [34]. Moreover, deletions and missense mutations of HIF-1α itself can increased its expression under nonhypoxic conditions by diminishing ubiquitination [35]. There were several HIF-1α isoforms existing in human benign tissues and malignancy [36]. It is curious that such Foxp3-related regulation is exclusive to full length HIF-1α but not to isoforms. In 293T cells with ectopic expression of full-length or truncated Foxp3 and HIF-1α protein, the C-terminus of Foxp3 can bind with N-terminus of HIF-1α [17]. Therefore, it is proposed that N-terminal domain variant (HIF1α1.2 or HIF1α1.3) and isoforms without exon 11 and 12 (HIF1α11-&12-, lack of proline hydroxylation) may escape from Foxp3-related regulation. For clinical implication, further study is required.

There were several limitations in our study. First, it is small and retrospective for the number of studied patients and specimens, so that we could not put all the significant variables into the same analysis in term of multivariate analysis. Second, for reducing the heterogeneity, we selected the published datasets according to the enough number of patients and same studied platform, there were only two datasets for meta-analysis. Third, unlike the other study with muscle-invasive bladder cancer [23], it is lack of correlation of Foxp3 expression with tumor stage or grade in the current cohort of 145 bladder tumors. Such an inconsistence may be caused by several reasons, including the studied subjects (the proportion of superficial *versus* muscle-invasive disease), the method of IHC evaluation, and statistics. On the other hand, both FGFR signaling and VEGF signaling can be regulated by HIF-1α expression in bladder cancer, which may reduce the correlation of Foxp3 expression with tumor stage and grade, because both of two signaling represent divergent molecular pathways in urothelial carcinogenesis [2]. Fourth, H-score method for evaluation of IHC study is not used in the current study because the studied tissue section is not a tissue microarray, so that the staining intensity may be not consistent among all the sections. For easy practice and statistic analysis, we utilized the method same with those used in the breast cancer by Merlo A et al [24].

In summary, Foxp3 expression is an unfavorable prognostic factor for disease progression in superficial bladder cancer patients, associated with less number of CD8^+^TILs. The *in vitro* model of T24 sublines showed Foxp3 upregulation is associated with increased aerobic glycolysis, GLUT member protein expression, and VEGF expression through decreasing HIF-1α protein degradation. These findings might provide Foxp3-targeted bladder cancer therapy and also some connection between tumor immunity and aerobic glycolysis.

## MATERIALS AND METHODS

### Cell lines and hypoxic circumstances, plasmids and trasfection

Three human bladder cancer cell T24 sublines were kindly provided by professor Hsieh, including T24-P (vector only), T24-L (primarily sub-cultured from lung metastatic site), and T24-B (primarily sub-cultured from bony metastatic site) [37]. All of three sublines were maintained in DME medium supplemented with 10% fetal bovine serum (FBS), 2 mM L-glutamine, and 50μg/ml gentamicin at 37°C in a humidified atmosphere of 5% CO_2_. Cells were seeded in 6- or 10-cm dishes overnight, refreshed the medium next day, and then placed in a hypoxia chamber (NexBiOxy) filled with 95% N_2_ and 5% CO_2_ to maintain O_2_ at 1% at the indicated periods. The hypoxic equipment was placed within a 37°C humidified incubator (Forma).

### Plasmids, trasfection and reagents

Two Foxp3 shRNAs were obtained from The RNAi Consortium (TRC) shRNA Library, including TRCN0000018959 and TRCN0000367825 (abbreviated as sh-Foxp3 #18 and #25). The plasmid pCMV3-N-HA-Foxp3 was purchased from Sino Biological Inc. (catalog number: HG11652-NY, Beijing, China). The plasmids pcDNA3.1-ubiquitin was obtained from Dr. Dimitris Xirodimas (University of Dundee, Scotland, UK). For plasmid transfection, the procotol is same with the previous study [38]. While cells were seeded in plates with 70-80% confluence, the transfection was carried out using polyethylenimine (PEI, Polysciences Inc., Warrington, PA) according to the manufacturer's instructions. Proteasome inhibitor MG132 was purchased from Calbiochem (Gibbstown, NJ).

### Quantitative RT-PCR, western blot analysis, and immunoprecipitation assay

Total ribonucleic acid (RNA) and cell lysates were harvested from T24 sublines at 80-90% confluence for further assays. Total RNA was extracted using the TRIzol® (Invitrogen, CA, USA) method according to the manufacturer's protocol and then reverse transcribed with High Capacity cDNA Reverse Transcription Kits (Applied Biosystems, CA, USA). The resulting cDNA was used for PCR in triplicate and data collections were performed in a Smart Cycler 2 PCR system (Cepheid, CA, USA). All samples were amplified simultaneously in duplicates in a one-assay run. The primers for human glucose transpoter members 1-5 (GLUT1-5) as well as for human VEGF_121_, and VEGF_165_ were shown in the supplementary information (Supplementary Table S2). The −ΔΔC_t_ method [39] was utilized to measure relative changes in mRNA levels examined by the quantitative reverse transcriptase PCR (qRT-PCR) experiments, after normalizing the transcript levels of each gene by the levels of β-actin as an internal control.

Western blotting and immunoprecipitation assays were performed as per our previous studies [40]. Thirty μg of protein from each sample was subjected to sodium dodecyl sulfate polyacrylamide gel electrophoresis (SDS-PAGE), transferred onto nitrocellulose membrane filter, and subsequently immunoblotted with anti-human/mouse Foxp3 purified antibody (clone eBio7979, eBioscience, CA, USA), anti-human Foxp3 (TA319911, Origene, Rockville, MD), and anti-human HIF-1α (category number, 610958; clone, 54/HIF-1α; BD Transduction Laboratories, San Jose, CA or GTX127309, Gentex). β-actin protein served as an internal control.

### Glucose, L-Lactate, and ATP assays

For measurements of ATP, glucose, and lactate in T24 sublines, three assay kits were utilized, including ATP Assay Kit (ab83355, abcam, MA, USA), Glucose Detection Kit (ab102517, abcam, MA, USA), and L-Lactate Assay Lit (ac65331, abcam, MA, USA). Cell lysates were harvested according to the manufactures' instructions and measured by enzymatically linking to the light reaction of bioluminescence enzymes, leading to light emission with the intensity being proportional to the tissue content of each metabolite.

### Immunofluorescence staining, and confocal microscopy

Cells grown on glass coverslips were fixed with 4% paraformaldehyde and permeabilized with 0.5% Triton X-100 and blocked with 5% BSA to reduce non-specific binding. After thorough washing, primary antibody was incubated overnight at 4°C. Antibody for human Foxp3 (TA319911, Origene, Rockville, MD) at 1:200 dilution or HIF-1α (BD Transduction Laboratories) at 1:400 dilution was incubated overnight at 4°C. Then, cells were incubated with either Alexa Fluor488 (mouse) or Alexa Fluor594 (rabbit)- conjugated secondary antibody (Invitrogen) at room temperature for 1 hour. DAPI was used for nuclear staining. The image analysis was performed by a FV1000 confocal microscope (Olympus) using 60×/1.4 NA oil objective lens (PLAPO). The images were adjusted using the Photoshop CS5 software (Adobe).

### *In vivo* ubiquitination assay

This assay was similar with our previous study [38]. In brief, the input fractions as indicated were prepared using RIPA buffer. His-tagged protein was pulled down using Dynabeads^®^ His-Tag Isolation and Pulldown kit (#10103D, Life technologies). Bound material was eluted from the beads, collected and subjected to SDS-PAGE and western blot analyses.

### *In vivo* tumor growth study

Female (NOD-SCID, 6-8 weeks old) mice were subcutaneously injected with 1×10^6^ Foxp3 knocking-down T24-B in 100 μl serum-free medium or its control. Tumor formation and growth were recorded every other day for at least 60 days according to the ‘Guidelines for the Welfare of Animals in Experimental Neoplasia’ (1998). Tumor volumes were calculated using the formula: length × (width)^2^ × 0.45. Once tumor burden grows more than 2500 mm^3^, it is viewed as death from tumor. At the end of this experiment, tumors were harvested for von Willebrand Factor immunostaining.

### Patient population and study samples

The study was undertaken with the approval and institutional oversight of the Institutional Review Board for the Protection of Human Subjects at both of Chia-Yi Christian Hospital (IRB-101014) and National Cheng Kung University Hospital (ER-95-49). Formalin-fixed paraffin-embedded tumor specimens from 145 bladder cancer patients were retrospectively retrieved from the archives of National Cheng Kung University Hospital and Christian Chia-Yi Hospital. All patients that received transurethral resection of bladder tumor (TURBT) and had been followed up for at least 1 year were included in the study. Tumors were staged according to the 2007 TNM classification and graded using the 2004 WHO classification. All the patients were managed according to the bladder cancer treatment guideline, modified from NCCN Clinical Practice Guidelines in Oncology. For superficial tumors, all patients received regular follow-up after intravesical therapy, including cystoscopy and surveillance of upper tract imaging. The regimens for intravesical therapy including either 40 mg epirubicin, 30 mg mitomycin-C weekly for 8 weeks, or 81 mg Bacillus Calmette-Guérin (BCG) vaccine for 6 weeks. Once the patient was diagnosed with muscle-invasive disease, either radical or partial cystectomy were suggested, and followed by adjuvant systemic chemotherapy with or without radiotherapy. The alternative choice is bladder sparing strategy with radical TURBT plus radiotherapy with or without systemic chemotherapy.

### Immunohistochemistry (IHC)

Serial 5-μm sections were cut for either hematoxylin and eosin (H & E) staining or IHC as per our previous study [40]. Briefly, after deparaffinization and rehydration, tissue sections were autoclaved and sequentially treated with 3% H_2_O_2_ in methanol, and then treated with skimmed milk in phosphate buffer saline. Nonspecific background staining was minimized by preincubating with 0.3% bovine serum albumin. Slides were incubated in the primary anti-Foxp3 antibody (clone 236/E7; ABCAM; dilution, 1:200) for 45 min, anti-CD8 monoclonal antibody (DAKO, Glostrup, Denmark; dilution, 1:200) for 1 hour, and polyclonal rabbit anti-human von Willebrand Factor (vWF) (DAKO, Glostrup, Denmark; dilution, 1:200). We performed parallel staining without primary antibody as a negative control, and human spleen section as a positive control. After incubation with secondary antibodies for 1 hour, the immunostaining was developed with a BioGenex Super sensitive Polymer HRP IHC System kit and then counterstained with hematoxylin. Samples were analyzed blindly by one pathologist.

Any tumor specimens exhibiting more than 25% immunoreactive tumor cells within one high power field were thought as FOXP3-positive tumors [24]. As for the significance of CD8-positive tumor-infiltrating lymphocytes (CD8^+^TILs), the average number was manually determined from 10 random 0.0328-mm^2^ digital images captured under high power field (×320). All counts were repeated 3 times by the same pathologist, and the average of the repeat counts was used for statistical analyses [27].

### Data mining with published datasets

For analyzing the correlation of Foxp3 expression with Glucose transporter and VEGF families, two human urothelial carcinoma dataset GSE32548 [41] and GSE48075 [42] were used for the used same platform (GPL6947, Illumina HumanHT-12 V3.0 expression beadchip) and larger number of patients (more than 100). Processed data were downloaded from NCBI GEO, and log2 data for individual probes were utilized for calculating the correlation coefficient between Foxp3 and GLUT and VEGF members using Pearson's method (Graphpad Software 6th version, San Diego, CA) and the metaanalysis (Comprehensive Meta-Analysis 2nd version, Biostat, Englewood, NJ, USA).

### Statistical analysis

The clinical outcomes of superficial diseases analyzed in the study were the first event of tumor recurrence in the urinary bladder and the first event of tumor progression into and beyond muscle layers. Recurrence-free survival (RFS) and progression-free survival (PFS) were calculated from the time of TUR-BT to the time of the first documented tumor recurrence in the urinary bladder, or tumor progression as muscle-invasive diseases, respectively. Those patients received TUR-BT with incomplete resection of tumor due to muscle invasiveness were viewed as clinical stage T2 at least and excluded from the survival analysis. Statistical analysis was performed using Statistical Package for Social Sciences, version 12.0, software (SPSS). The relationships between the presence of Foxp3-positive tumors, clinicopathological factors, and clinical outcome were analyzed with Kaplan-Meier plots, the log-rank test, and the multivariate Cox regression model. All *p* values reported were two-sided and considered significant if *p* < 0.05.

## SUPPLEMENTARY FIGURES AND TABLES


