# Supplementary material for: Foxp3 enhances HIF-1α target gene expression in human bladder cancer through decreasing its ubiquitin-proteasomal degradation
Source: Oncotarget. 2016 Aug 19;7(40):65403–17. doi: 10.18632/oncotarget.11395 (PMC5323164; doi:10.18632/oncotarget.11395)
Supplement: Supplementary file 1 [file oncotarget-07-65403-s001.pdf]

Foxp3 enhances HIF-1α target gene expression in human bladder cancer through decreasing its ubiquitin-proteasomal degradation

SUPPLEMENTARY FIGURES AND TABLES

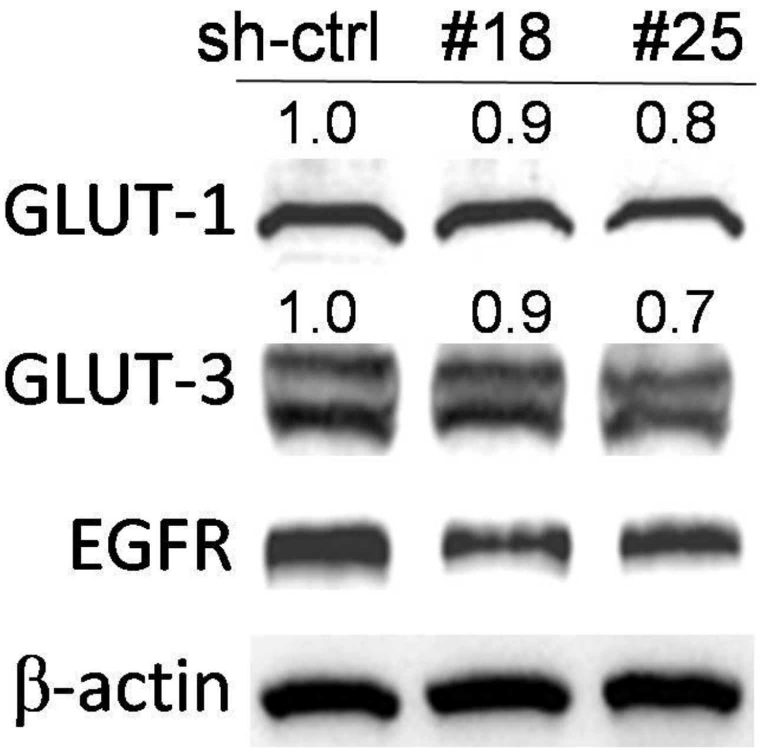

Supplementary Figure S1: Knocking-down of Foxp3 expression decreased HIF-1α target gene expression, such as GLUT-1, -3, and EGFR.

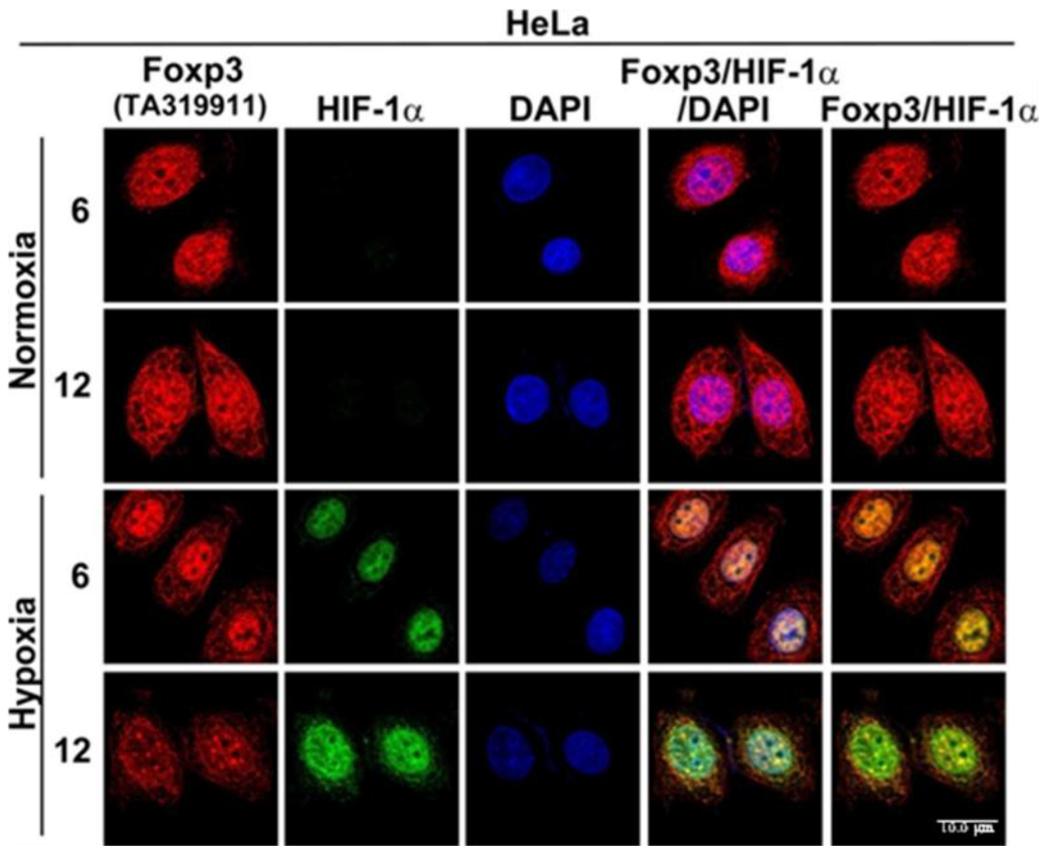

Supplementary Figure S2: HeLa cells were maintained in normoxic and hypoxic circumstance at the indicated times. Cell were stained with anti-Foxp3, anti-HIF-1α and DAPI for confocal microscopy studies.

Supplementary Table S1: Clinicopathological correlate of Foxp3 expression in human bladder carcinoma tissues

	Total	Fox p3(-)	Fox p3(+)	*p* value
Total, n	145	112	33	
Age, (yr), mean ± SD	67.5 ± 12.3	67.6 ± 12.8	67.2 ± 10.3	0.864
Median	69	70	66	
25~75 percentile	59~76	57~77	60~75	
Gender				
Male	98	82	16	0.008
Female	47	30	17	
History of UC				
Primary	84	66	18	0.654
Recurrence	61	46	15	
Multiplicity				
Single	49	37	12	0.722
Multiple	96	75	21	
Morphology				
Papillary	112	84	28	0.236
Non-papillary	33	28	5	
Tumor grade				
Low	17	12	5	0.610
High	128	90	28	
Stage				
T0	56	43	13	0.645
T1	59	44	15	
T2 at least	30	25	5	

UC, urothelial carcinoma

Supplementary Table S2: Primers for glucose transporter 1-5

Member	Forward/Reverse	Primer sequence
GLUT-1	Forward	TAAGGACACACTAATCGAACTATGAACT
	Reverse	GGTCTCAGGTAAAGAAAGATTAATTTGA
GLUT-2	Forward	TAAATGTTCAGCTTCAGACTTTTATCAA
	Reverse	GTAAAGGCAGATAGATAGTGTACAATGC
GLUT-3	Forward	TTCTGGCTCCTCAAACAGTAGGTTGGCA
	Reverse	TCCATATGGAAATATTCACAATCTTCTC
GLUT-4	Forward	ACTTCTATAAAGTCACTGCTGAAGACAA
	Reverse	AGCTACAATTTATTGAGCATTTACTATT
GLUT-5	Forward	GGCTTCTCCATCTGCCTCATAG
	Reverse	GGAGATGACACAGACGATGCTG
$VEGF_{121}$	Forward	GCAGAATCATCACGA AGTGG
	Reverse	GCATGGTGATGTTGGACTCC
$VEGF_{165}$	Forward	ATCTTCAAGCCATCCTGTGTGC
	Reverse	CAAGGCCCACAGGGATTTTC
